# Manipulation of PBF/PTTG1IP Phosphorylation Status; a Potential New Therapeutic Strategy for Improving Radioiodine Uptake in Thyroid and Other Tumors

**DOI:** 10.1210/jc.2012-3640

**Published:** 2013-05-15

**Authors:** V. E. Smith, N. Sharma, R. J. Watkins, M. L. Read, G. A. Ryan, P. P. Kwan, A. Martin, J. C. Watkinson, K. Boelaert, J. A. Franklyn, C. J. McCabe

**Affiliations:** School of Clinical and Experimental Medicine (V.E.S., N.S., R.J.W., M.L.R., G.A.R., P.P.K., J.C.W., K.B., J.A.F., C.J.M.), Institute of Biomedical Research, and School of Cancer Sciences (A.M.), University of Birmingham, Birmingham B15 2TT, United Kingdom

## Abstract

**Context:**

The clinical effectiveness of ablative radioiodine treatment of thyroid tumors is limited by the availability of the sodium iodide symporter (NIS) at the plasma membrane (PM) for uptake of ^131^I. A significant proportion of well-differentiated thyroid tumors are unable to concentrate sufficient radioiodine for effective therapy, and in other tumor models such as breast tumors, where radioiodine uptake would be an attractive therapeutic option, uptake is insufficient.

**Objective:**

Pituitary tumor–transforming gene-binding factor (PBF; PTTG1IP) is overexpressed in multiple cancers and significantly decreases NIS expression at the PM. The goal of this study was to identify a method by which PBF repression of NIS may be overcome in human tumors.

**Results:**

Here, we identify PBF as a tyrosine phosphoprotein that specifically binds the proto-oncogene tyrosine protein kinase Src in mass spectrometry, glutathione *S*-transferase pulldown and coimmunoprecipitation assays. Src induction leads to phosphorylation at PBF residue Y174. Abrogation of this residue results in PM retention and a markedly reduced ability to bind NIS. The Src inhibitor PP1 inhibits PBF phosphorylation in multiple cell lines in vitro, including human primary thyroid cells. Of direct clinical importance to the treatment of thyroid cancer, PP1 stimulates iodide uptake by transfected NIS in TPC1 thyroid carcinoma cells and entirely overcomes PBF repression of iodide uptake in human primary thyroid cells.

**Conclusions:**

We propose that targeting PBF phosphorylation at residue Y174 via tyrosine kinase inhibitors may be a novel therapeutic strategy to enhance the efficacy of ablative radioiodine treatment in thyroid and other endocrine and endocrine-related tumors.

Radioiodine is a safe and effective modality used for more than 60 years in the treatment of thyroid cancer and has potential uses in breast, prostate, and other cancers. Ablative radioiodine therapy exploits the ability of the thyroid to take up iodide, a process mediated by the sodium iodide symporter (NIS), which transports iodide across the basolateral plasma membrane (PM) of thyroid follicular epithelial cells for thyroid hormone biosynthesis (1, 2). Many thyroid cancers demonstrate reduced NIS activity through diminished expression (3) and intracellular retention (4–6). TSH can induce both NIS expression and cell surface targeting, and TSH stimulation is required to successfully induce iodide uptake in most well-differentiated thyroid tumors. However, up to 20% of these tumors fail to concentrate enough radioiodine for effective therapy, even after TSH stimulation (3). Further, dedifferentiated and metastatic thyroid tumors generally respond poorly to radioiodine therapy. Hence, tumors with reduced NIS activity are commonly associated with a poor prognosis. More broadly, whereas the endogenous NIS expression demonstrated in breast tumors can facilitate radioiodine uptake (6, 7) and multiple tumors can be engineered to do so, uptake in these cancers is currently inadequate for effective therapeutic efficacy (8).

Also known as PTTG1IP or c21orf3 (9), pituitary tumor–transforming gene-binding factor (PBF) is transforming in vitro, is tumorigenic in vivo (10), and is induced in breast tumors (11). In thyroid cancer, PBF is significantly up-regulated, and higher PBF expression is independently associated with early tumor recurrence and an overall poorer disease outcome (10, 12, 13). We have previously implicated PBF in 2 discrete mechanisms of NIS repression (14–16). PBF overexpression has been demonstrated to repress NIS expression both in vitro and in vivo (14, 16). Further, PBF binds to NIS and reduces iodide uptake through modulation of its subcellular localization (15). In addition, the thyroid hormone transporter MCT8, which mediates the secretion of thyroid hormone, was recently found to be similarly regulated by PBF, suggesting that PBF may be a key regulator in thyroid hormone biosynthesis and secretion (17). Indeed, thyroid-specific PBF overexpression in the mouse resulted in gross thyroid gland enlargement, hyperplasia, macrofollicular lesions, and, significantly, a greatly impaired ability for uptake of radioiodine (16). PBF is, thus, a protein that is dysregulated in thyroid tumorigenesis and that elicits potent repression of NIS function.

Understanding the factors repressing NIS activity is critical in improving radioiodine delivery to tumors that are unable to take up radioiodine effectively. Intensive interest therefore exists in identifying mechanisms of up-regulating endogenous NIS expression through the use of histone deacetylase inhibitors, demethylating agents, nuclear receptor agonists, and kinase inhibitors and in tumor-targeted NIS gene therapy (18). However, our data suggest that if PBF is overexpressed in a tumor, induction of NIS expression alone may be insufficient, given the posttranslational repression of NIS by PBF (15). Thus, a therapeutic strategy targeting PBF would also be needed for increased clinical effectiveness.

In the current investigation, we have extended our initial observation that PBF overexpression can posttranslationally repress NIS in normal thyroid cells by demonstrating NIS and PBF colocalization within the intracellular vesicles of multiple human tumor cell lines. These included papillary and anaplastic thyroid, breast, and prostate cancer cells. We have determined that PBF is phosphorylated at tyrosine 174 (Y174), a residue we have found to be important in the regulation of PBF endocytosis. PBF bound to the tyrosine kinase Src, with induced Src expression, resulting in increased PBF Y174 phosphorylation. Conversely, the Src inhibitor PP1 reduced pY174 levels in multiple cell lines and in human primary thyroid cells. Abrogation of Y174 significantly diminished the interaction between PBF and NIS, and pY174 PBF colocalized with NIS. Critically, treatment with PP1 overcame PBF repression of iodide uptake into human primary thyroid cells. We suggest therefore that targeting PBF phosphorylation may have a significant impact on the efficacy of radioiodine treatment of thyroid and other tumors, where uptake is currently insufficient for effective ablation.

## Materials and Methods

### Cell lines and human primary thyroid culture

COS-7 African green monkey kidney epithelial, HeLa human cervical carcinoma, and T47D human breast cancer cell lines were maintained in DMEM, high glucose (PAA, Pasching, Austria). TPC1, K1, and SW1736 thyroid carcinoma, Saos-2 osteosarcoma, LNCaP prostate cancer, and A2780 ovarian carcinoma cell lines were maintained in RPMI 1640 medium (Life Technologies, Inc, Paisley, Scotland, United Kingdom). VCaP prostate cancer and HCT166 colorectal carcinoma cell lines were maintained in DMEM/F-12 and McCoy's 5A media (Life Technologies, Inc), respectively. All were supplemented with 10% fetal bovine serum (FBS), penicillin (10^5^ U/L), and streptomycin (100 mg/L).

Collection of thyroid samples was in accordance with approval of the local research ethics committee, and subjects gave informed written consent. Preparation of human thyroid follicular cells from surgical specimens was based on a method described previously (19). In brief, thyroid tissue was digested using 0.2% collagenase (Worthington Biochemical Corp, Lakewood, New Jersey). Follicles were plated in medium described by Ambesi-Impiombato et al (20), supplemented with TSH (300 mU/L), insulin (300 μg/L), penicillin (10^5^ U/L), streptomycin (100 mg/L), and 4% FBS. After 72 hours, FBS was omitted, and experiments were performed ∼11 days later.

### Plasmids and transfection

Plasmids containing the full-length PBF cDNA with a hemagglutinin (HA) tag and NIS cDNA with a MYC tag have been described previously (10, 15). Mutants Y174A and F177A were created by mutating the PBF-HA plasmid using a QuikChange Site-Directed Mutagenesis Kit (Stratagene, Cambridge, United Kingdom) with the primers 5′-CCTGTTTAAAGAAGAAAACCCGGCTGCTAGATTTGAAAACAACTAC-3′ and 5′-GAAAACCCGTATGCTAGAGCTGAAAACAACTACCCATACG-3′, respectively. Nucleotide substitutions are underlined. Full-length Src cDNA (kindly donated by Dr. Yotis Senis, University of Birmingham, Birmingham, United Kingdom) was subcloned into pcDNA3.1+ (Life Technologies, Inc) using the *Eco*R1 and *Xba*1 restriction sites.

Cells were transfected using FuGENE 6 reagent (Roche, Indianapolis, Indiana), following the manufacturer's instructions at a 3:1 reagent to DNA ratio.

### Antibodies and reagents

The following antibodies were used: mouse monoclonal anti-HA.11 antibody (Covance Research Products, Princeton, New Jersey), rabbit polyclonal anti-HA (Y-11) antibody (Santa Cruz Biotechnology, Santa Cruz, California), PBF phospho-specific antibody to residue Y174 (CovalAb, Villeurbanne, France), mouse monoclonal anti-Myc-Tag (9B11) antibody (Cell Signaling Technology, Danvers, Massachusetts), rabbit anti-Src (36D10) antibody (Cell Signaling Technology), rabbit polyclonal anti-PBF antibody (made by Eurogentec [Seraing, Belgium] for our laboratory using the full-length PBF protein as an epitope), rabbit polyclonal anti-NIS antibody (ab104920; Abcam, Cambridge, United Kingdom), and mouse monoclonal anti–β-actin antibody (clone AC-15; Sigma, Poole, Dorset, United Kingdom). The Src kinase inhibitor PP1 and the negative control compound PP3 were kind gifts from Dr Yotis Senis.

### Immunofluorescence staining

Immunofluorescence staining was performed as described previously (15). For detection of pY174, cells were treated for 15 minutes with pervanadate (100 μM) before fixation.

Epifluorescent microscopy was performed using ×40 and ×100 objectives on a Zeiss Axioplan fluorescent microscope (Zeiss, Oberkochen, Germany). A Zeiss confocal LSM 510 microscope with ×63 and ×100 objectives was used to perform confocal microscopy.

### Western analysis

Western blotting was performed throughout as described previously (17). For assessment of pY174 expression, cells were treated for 15 minutes with pervanadate (100 μM) before lysis in a modified radioimmunoprecipitation assay buffer (50 mM Tris-HCl [pH 7.4], 150 mM NaCl, 1% vol/vol Igepal CA-630, 6 mM sodium deoxycholate, and 1 mM EGTA) containing protease inhibitor cocktail (Sigma) and 1 mM sodium orthovanadate (Sigma). PBF-HA or β-actin expression was assessed to determine total PBF expression and/or act as a loading control where appropriate.

### Cell surface biotinylation

PBF-HA, Y174A, and F177A plasmids (2 μg) were transfected into COS-7 cells in 6-well plates. Cell surface biotinylation assays were performed as described previously (17). Wild-type and mutant PBF were subsequently detected in both membrane protein and whole-cell lysate fractions by Western analysis using mouse anti-HA.11 antibody (Covance Research Products).

### Immunoprecipitation/coimmunoprecipitation assays

All transfections for these assays were performed in T25 flasks using 5 μg of DNA. Immunoprecipitation (IP) assays were performed as described previously (17).

To determine specific binding of the pY174 antibody, PBF-HA and Y174A were transfected into COS-7 and K1 cells and immunoprecipitated from lysate treated for 15 minutes with pervanadate (100 μM) before being harvested with mouse anti-HA.11 antibody (Covance Research Products). Phosphorylated PBF was detected after Western blotting and probing with the phospho-specific pY174 antibody.

To assess a possible interaction between PBF and Src, HeLa cells were transfected with PBF-HA and vector only (VO) control, PBF-HA and Src, and VO and Src. After IP of PBF-HA with mouse anti-HA.11 antibody (Covance Research Products), the coimmunoprecipitation of exogenous Src was determined by Western blotting and probing with rabbit anti-Src (36D10) antibody (Cell Signaling Technology). The reciprocal assay was also performed.

To assess the effect of the PBF mutation on binding to NIS, coimmunoprecipitation assays were performed as described previously with lysate from COS-7 cells transfected with NIS-MYC and either VO, PBF-HA, or Y174A (15).

### Mass spectrometry

After transfection with PBF-HA, K1 and TPC1 cells were lysed in high-salt lysis buffer (50 mM Tris-HCl [pH 7.4], 400 mM NaCl, and 1% vol/vol Igepal CA-630). Proteins interacting with PBF-HA were isolated through coimmunoprecipitation using mouse anti-HA.11 antibody (Covance Research Products) and separated by SDS-PAGE. Gels were Coomassie-stained, and each lane was excised and divided into approximately 20 2-mm segments. After destaining, proteins were reduced, alkylated, and trypsinized before undergoing HPLC using an acetonitrile gradient. Samples were then passed straight through an amaZon ETD ion trap and tandem mass spectrometer (Bruker Daltronics, Coventry, United Kingdom).

### In vitro transcription: translation and glutathione *S*-transferase (GST) pulldown assays

The TNT-coupled reticulocyte lysate system (Promega, Madison, Wisconsin) was used to express l-α-[^35^S]methionine–labeled protein from the Src plasmid. GST pulldown assays were performed to assess binding between GST-tagged PBF (GST-PBF) and l-α-[^35^S]methionine–labeled Src as described previously (17).

### Proximity ligation assay (PLA)

The Duolink In Situ Kit (Olink, Uppsala, Sweden) was used to detect protein-protein interactions. COS-7 cells were transfected with NIS-MYC and PBF-HA and treated 48 hours later with pervanadate (100 μM) for 15 minutes before fixation. To assess Src inhibition, cells were treated for 24 hours with either PP1 (2 μM) or an equivalent volume of dimethyl sulfoxide (vehicle). Fixation, permeabilization, blocking, and primary antibody (mouse anti-myc and either rabbit anti-HA or rabbit anti-PBF-pY174) incubations were as performed in the immunofluorescent studies. The Duolink In Situ Kit was then used as per the manufacturer's instructions. Images were obtained using the Zeiss confocal LSM 510 microscope ×40 objective.

### Iodide uptake assays

In 24-well plates, TPC1 cells were transfected with either VO or NIS-HA, and human primary thyroid cultures were transfected with VO or PBF-HA (0.5 μg/well). Cells were treated at 24 hours posttransfection with either PP1 (2 μM) or an equivalent volume of dimethyl sulfoxide (vehicle), and after a further 24 hours, uptake assays were performed. To demonstrate specific uptake in the TPC1 cells, the NIS inhibitor sodium perchlorate was used at 100 μM to pretreat control wells for 1 hour before the addition of ^125^I. NaI (final concentration 10 μM) and 0.1 μCi of ^125^I were added directly to the TPC1 cell medium, and 1 μM NaI and 0.05 μCi of ^125^I were added to the primary thyroid culture medium. After incubation at 37°C of the TPC1 and primary thyroid cells for 1 and 3 hours, respectively, medium was removed, and cells were washed rapidly with Hanks' balanced salt solution. Cells were lysed in 2% sodium dodecyl sulfate, and the radioactivity of the lysate was counted for 1 minute in a gamma counter. Results are expressed as picomoles of I^−^ per microgram of protein.

### Statistical analyses

Data were analyzed using SigmaStat (SPSS Science Software Ltd, Birmingham, United Kingdom) and are displayed as means ± SEM. All statistical tests were performed with either the Student's *t* test or Mann-Whitney rank sum test. Significance was taken as *P* < .05.

## Results

### Mutation of a putative tyrosine sorting signal results in PBF accumulation at the PM

**Figure 1.**
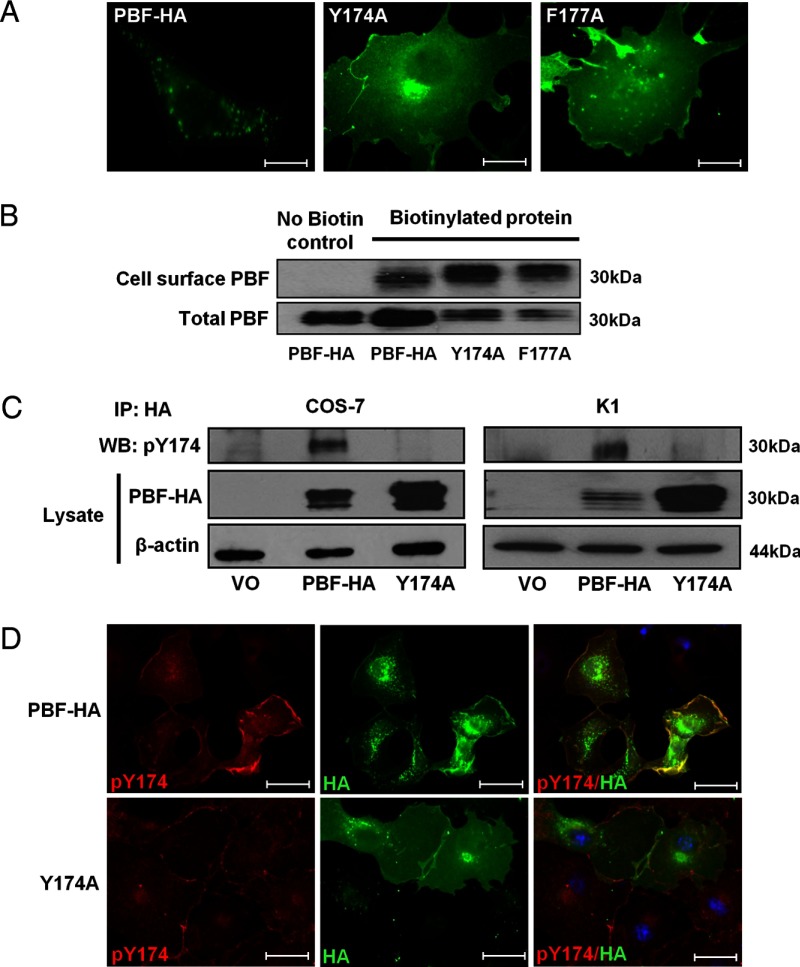
PBF is phosphorylated at tyrosine residue 174, which is critical for endocytosis. A, Subcellular localization of HA-tagged wild-type and mutant PBF was determined by immunofluorescent staining using an anti-HA antibody after transfection into COS-7 cells. Both Y174A and F177A accumulated at the plasma membrane, in contrast to PBF-HA, which was localized mainly in intracellular vesicles. Bars, 20 μm. B, Representative cell surface biotinylation assay demonstrating increased plasma membrane expression of the Y174A and F177A mutants compared with that for PBF-HA. PBF is a glycoprotein that is typically detected with bands of between 25 and 30 kDa. C, IP of PBF-HA and subsequent probing with a phospho-specific pY174 antibody in COS-7 and K1 cell lysate, confirming detection of a specific product at the predicted molecular mass but not the Y174A mutant. D, Immunofluorescent studies in COS-7 cells transfected with PBF-HA and Y174A and probed with our phospho-tyrosine antibody and an anti-HA antibody. Coincident expression (yellow) was apparent for tyrosine-phosphorylated PBF (red) and exogenous PBF (green) for PBF-HA but not the Y174A mutant. Bars, 20 μm.

To identify a way to disrupt the inhibitory effect of PBF on NIS, we initially sought to increase our understanding of PBF cellular trafficking. Deletion of the C-terminal region of PBF (residues 149–180) has been shown previously to increase PM localization (15). Given that PBF localized within late endosomes with the tetraspanin CD63 (15), which is commonly associated with clathrin-dependent endocytosis, we hypothesized that this was due to the loss of a putative tyrosine-based sorting signal (YXXΦ) and therefore investigated the functional consequence of the specific mutation of this motif. Substitution of the critical tyrosine (Y174A) and hydrophobic (F177A) residues resulted in PBF accumulation at the PM, in contrast to the largely vesicular localization of wild-type HA-tagged PBF (PBF-HA), as demonstrated using immunofluorescent studies (Figure 1A). Cell surface biotinylation assays provided additional confirmation (Figure 1B). Thus, discrete abrogation of either the Y174 or F177 residue resulted in increased PM retention, confirming the presence of a functional YXXΦ internalization motif.

### The key tyrosine residue within the sorting signal (Y174) is phosphorylated

Within the YXXΦ internalization motif, the critical tyrosine residue at amino acid 174 is strongly predicted to be a site of phosphorylation (www.phosphosite.org) (21). Because phosphorylation of this residue would impair its interaction with clathrin-associated adaptor complexes (22), such a modification may regulate PBF localization. To explore this hypothesis we constructed a phospho-specific antibody to residue Y174. Initially, IP of PBF-HA and subsequent probing with our pY174 antibody confirmed detection of a specific product at the predicted molecular mass (Figure 1C) in COS-7 cells, in which we have previously examined PBF function, as well as papillary thyroid carcinoma K1 cells. The Y174A mutant, however, was not detected by the phospho-specific antibody. Immunofluorescent staining of PBF-HA–transfected COS-7 cells with our pY174 antibody revealed coincident expression of tyrosine-phosphorylated PBF and total exogenous PBF, particularly within the PM (Figure 1D). Further, in Y174A-transfected cells, the pY174 antibody detected endogenous phospho-PBF at the PM but not the Y174A mutant, confirming specificity of the antibody (Figure 1D). Phospho-specific detection of pY174 was further established through experiments performed in the presence and absence of the protein tyrosine phosphatase inhibitor pervanadate (see Supplemental Figure 1 published on The Endocrine Society's Journals Online web site at http://jcem.endojournals.org.).

### PBF colocalizes with NIS in numerous cell lines

**Figure 2.**
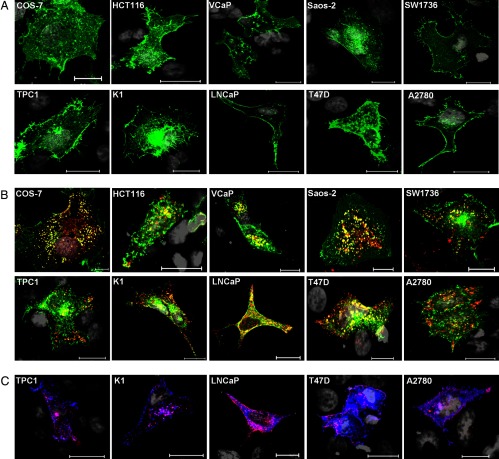
PBF and NIS colocalization in multiple cell lines. A, Confocal immunofluorescence microscopy demonstrating MYC-tagged NIS (green) localization in COS-7, HCT116, VCaP, Saos-2, SW1736, TPC1, K1, LNCaP, T47D, and A2780 cells. Bars, 20 μm. B, Confocal microscopy demonstrating PBF-HA (red) and NIS-MYC (green) expression, detected using anti-HA and anti-MYC antibodies, respectively, with specific colocalization (yellow) observed predominantly within intracellular vesicles. Bars, 20 μm. C, Confocal images of PBF pY174 (red) and NIS-MYC (blue) localization, determined using anti-pY174 and anti-MYC antibodies, respectively, in TPC1, K1, LNCaP, T47D, and A2780 cells. Specific colocalization is represented in magenta. Bars, 20 μm.

PBF is a transmembrane protein that shuttles NIS between the PM and the cytoplasm, with profound implications for ablative radioiodine uptake during thyroid cancer treatment (15). We have previously demonstrated PBF colocalization with NIS in COS-7 and FRTL-5 rat thyroid cells, predominantly within intracellular vesicles of the late endosome phenotype (15). We now extend this observation to 9 commonly used cancer cell lines of breast (T47D), prostate (VCaP and LNCaP), colorectal (HCT116), ovarian (A2780), and osteosarcoma (Saos-2) lineages, as well as K1, TPC1, and SW1736 thyroid cells. Relative endogenous expression of PBF was characterized in a number of these cell lines as determined by Western analysis (Supplemental Figure 2). MYC-tagged NIS was initially transfected alone and appeared to be correctly targeted to the PM in each of these cell lines (Figure 2A). Variable amounts of intracellular expression were also observed. After cotransfection, PBF-HA showed vesicular colocalization with NIS-MYC in each of these cell lines (Figure 2B), with varying degrees of PM colocalization, as we have described previously for COS-7 cells (15). On this basis, we consider that PBF colocalization with NIS is broadly universal.

Given that PBF is phosphorylated at Y174 and colocalizes with NIS, we next determined through confocal microscopy whether the pY174 form of PBF localizes with NIS in a subset of this collection of cell lines. Compared with total PBF immunofluorescence (Figure 2B) and consistent with the images in COS-7 cells (Figure 1D), PBF pY174 appeared to be distributed more readily at the PM in these cells (Figure 2C). Specific colocalization of NIS-MYC and PBF pY174 (magenta) was evident, both within intracellular vesicles and at the PM (Figure 2C). In contrast, significant intracellular colocalization was not evident between NIS-MYC and the Y174A mutant (Supplemental Figure 3).

### PBF interaction with the tyrosine kinase Src

**Figure 3.**
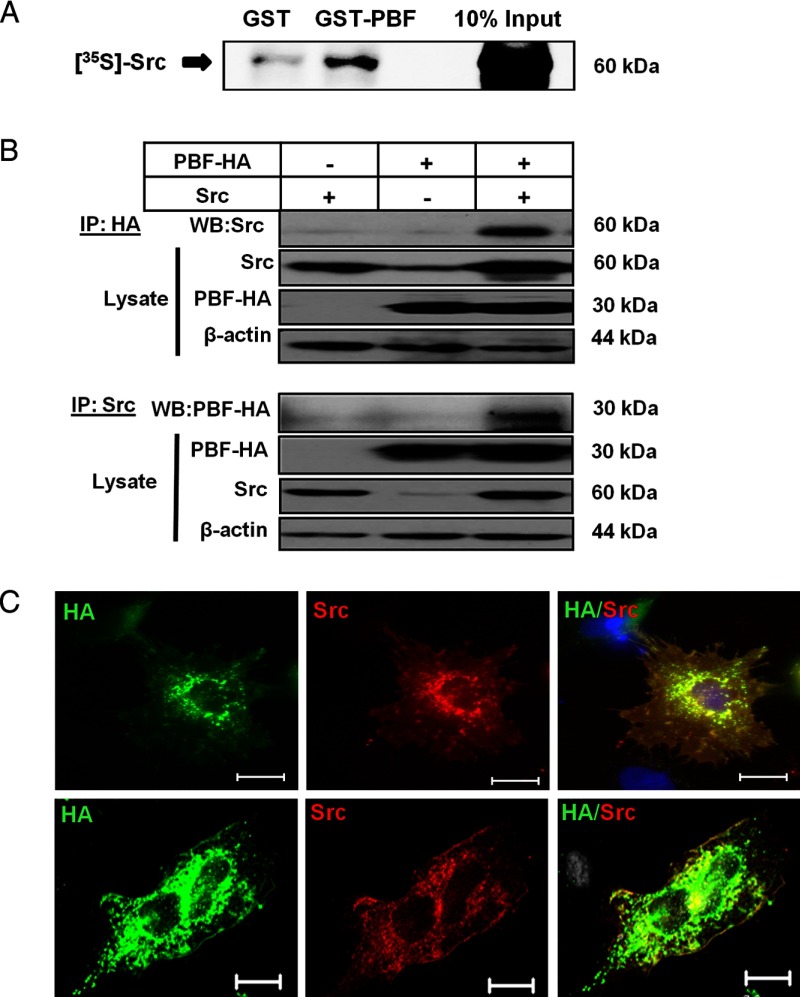
Src interaction and colocalization with PBF. A, GST pulldown assay demonstrating in vitro binding between GST-tagged PBF (GST-PBF) and l-α-[^35^S]methionine–labeled Src. GST-only and 10% input lanes are shown for comparison. B, After IP of PBF-HA with anti-HA antibody from HeLa cell lysate, the coimmunoprecipitation of exogenous Src was determined by Western blotting and probing with anti-Src antibody. The reciprocal assay is also shown. WB, Western blot. C, Epifluorescent (top panel) and confocal (bottom panel) images of PBF-HA (green) and Src (red) localization, determined using anti-HA and anti-Src antibodies, respectively, with specific colocalization (yellow) in HeLa cells. Bars, 20 μm.

Because abrogation of Y174 altered PBF subcellular localization (Figure 1A) and phospho-PBF colocalized with NIS (Figure 2C), we studied the pY174 modification in further detail and examined whether this might regulate the ability of PBF to alter NIS localization. To screen for a tyrosine kinase capable of phosphorylating PBF, we performed extensive tandem mass spectrometry studies in different cell lines. These studies identified the proto-oncogene tyrosine protein kinase Src as a putative binding partner of PBF (peptide match with a score of 26.8 and sequence coverage of 6.5%). On this basis, we performed GST pulldown assays in which GST-tagged PBF pulled back l-α-[^35^S]methionine–labeled Src (Figure 3A). We therefore proceeded to coimmunoprecipitation assays in HeLa cells to examine putative binding in a cellular context. Coimmunoprecipitated Src was detected after the IP of PBF-HA with a HA antibody (Figure 3B). In the reciprocal assay, IP of Src and the subsequent detection of coimmunoprecipitated PBF-HA further established an in vitro interaction between PBF and Src (Figure 3B).

The subcellular localization of this interaction in vitro was assessed through immunofluorescent analysis of HeLa cells cotransfected with PBF-HA and Src. Colocalization of PBF-HA and Src was observed predominantly at the PM and in the perinuclear region (Figure 3C). Given the analogous staining pattern of pY174 (Figure 2B), these data further suggest that Src binds and phosphorylates PBF and that this occurs either at the PM, in the perinuclear region, or both.

### Effect of manipulating Src on pY174

**Figure 4.**
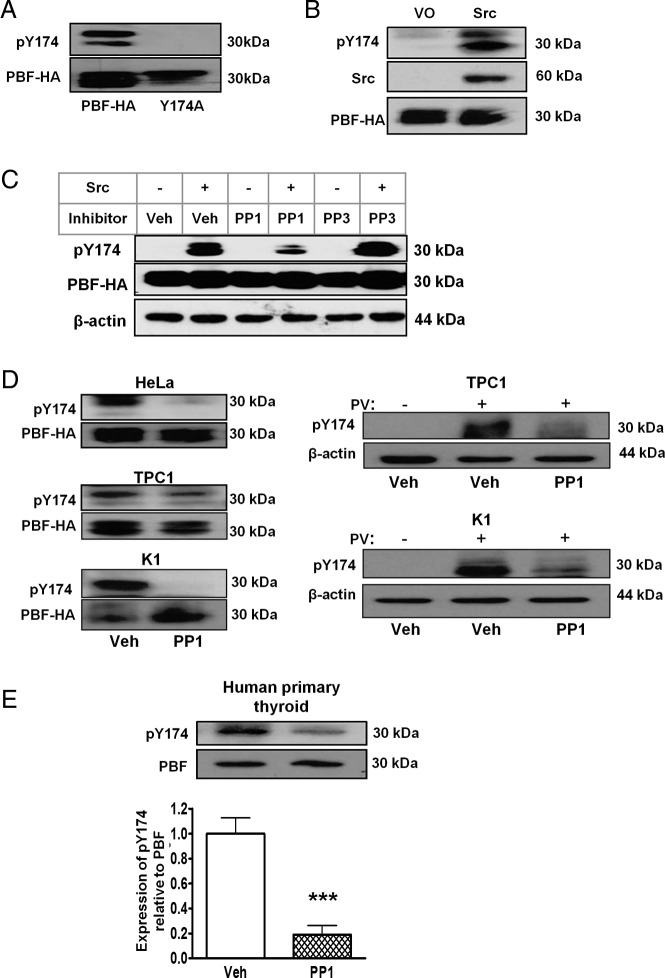
Src regulates Y174 phosphorylation. A, Western analysis of pY174 in lysate from HeLa cells transfected with PBF-HA and Y174A. PBF is a glycoprotein that is typically detected with bands of between 25 and 30 kDa. B, HeLa cells transfected with PBF-HA and Src demonstrated significantly increased pY174 compared with PBF-HA and VO controls. C, Treatment of HeLa cells transfected with PBF-HA and Src with 2 μM Src kinase inhibitor PP1 for 30 minutes elicited potent repression of pY174 expression compared with that for vehicle only treatment (Veh), whereas the related negative control compound PP3 (2 μM) failed to alter pY174 phosphorylation. D, PP1 treatment (2 μM for 30 minutes) resulted in the inhibition of pY174 in HeLa cells and the papillary carcinoma thyroid lines TPC1 and K1 transfected with PBF-HA. Right-hand panel, endogenous pY174 expression in TPC1 and K1 cells observed with pervanadate (PV) treatment and inhibited by PP1 treatment (2 μM for 24 hours). E, Endogenous pY174 in human primary thyroid cultures treated with 2 μM PP1 for 30 minutes (each treatment was performed in duplicate in each of n = 5 preparations). ***, *P* < .001.

To determine whether Src is indeed capable of phosphorylating PBF, we transfected HeLa cells with PBF-HA and Src and examined pY174 expression. Western blotting confirmed detection of pY174 in HeLa cells transfected with PBF-HA that was absent in Y174A-transfected cells (Figure 4A). Subsequently, transient overexpression of Src resulted in significantly increased phosphorylation of PBF-HA compared with VO controls (Figure 4B). To determine whether this was reversible, we appraised the Src kinase inhibitor PP1, along with a negative control compound PP3, which shares a structure similar to that of PP1 but does not affect Src kinase activity. Compared with vehicle, PP1 resulted in a potent repression of Src-induced pY174 expression. In contrast, parallel treatment with PP3 failed to alter Src-induced pY174 phosphorylation (Figure 4C). Suppression of pY174 by PP1 was also observed without Src overexpression in HeLa cells transfected with PBF-HA (Figure 4D).

Activated Src has been demonstrated in a number of thyroid carcinoma cell lines (23), and we assessed PBF phosphorylation in the papillary carcinoma thyroid lines TPC1 and K1. After transfection of PBF-HA in TPC1 and K1 cell lines, pY174 was both detected and highly sensitive to PP1 treatment (Figure 4D). Notably, endogenous pY174 expression could also be detected and inhibited by PP1 treatment in TPC1 and K1 thyroid cell lines (Figure 4D). Given that PBF function has been reported to be central to iodide uptake and thyroid hormone efflux in thyroid cells (15, 17), we further extended these observations to human primary thyroid cultures. Despite the reported heterogeneity of such cultures, multiple preparations demonstrated endogenous PBF pY174 phosphorylation, which was significantly inhibited by PP1 (81.1 ± 7.8% reduction, *P* < .001, n = 5) (Figure 4E). These data demonstrate that Src overexpression induces pY174 and Src inhibition suppresses pY174. Interestingly, endogenous Src-mediated pY174 is observed in human primary thyroid cultures, suggesting this may be a mechanism of PBF regulation apparent in normal thyroid cells.

### The Y174 site is important for NIS interaction

**Figure 5.**
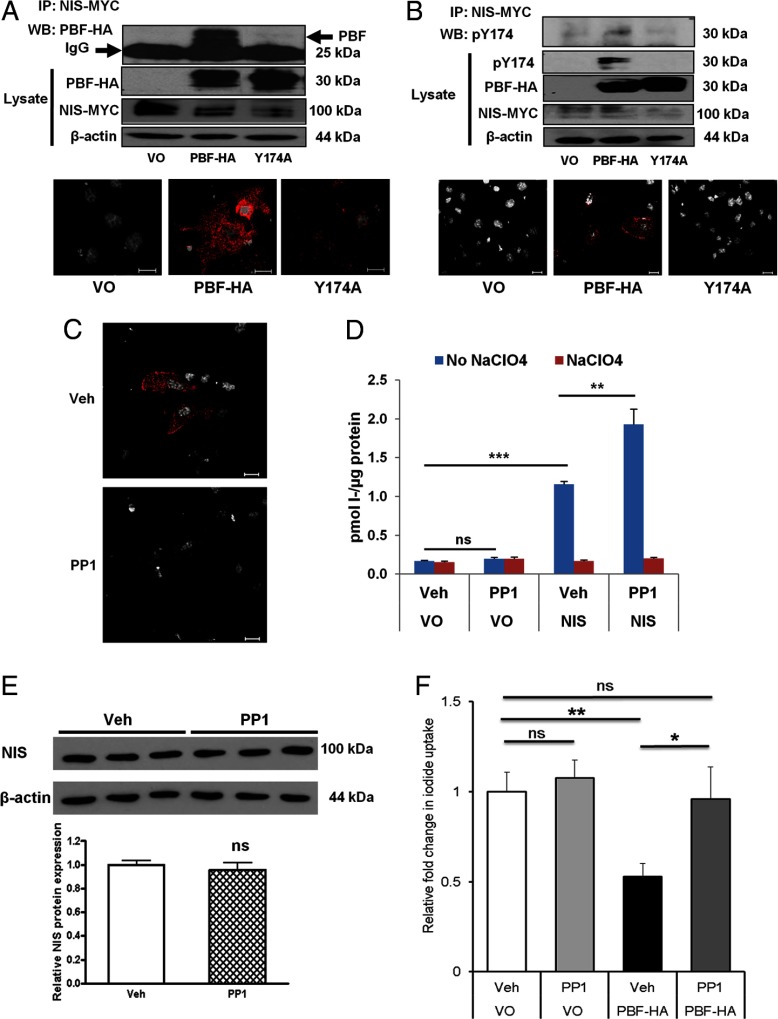
Y174 is critical for NIS binding, and Src inhibition overcomes PBF repression of iodide uptake. A, Coimmunoprecipitation assay in COS-7 cells. After IP of NIS-MYC with a mouse anti-Myc antibody, PBF-HA was detected using mouse anti-HA antibody. Y174A substitution diminished interaction with NIS. IgG, light chain immunoglobulin. WB, Western blot. Bottom panel, representative images of a PLA showing an interaction between NIS-MYC and PBF-HA in COS-7 cells using anti-MYC and anti-HA antibodies. Specific interactions are visualized with a red fluorescent spot. B, Similarly, after IP of NIS-MYC from HeLa cell lysate, an interaction with PBF-pY174 was detected using the pY174 antibody. Bottom panel, representative images of a PLA showing an interaction between NIS-MYC and PBF-pY174 in COS-7 cells using anti-MYC and anti-pY174 antibodies. C, Protein-protein interactions between PBF-pY174 and NIS-MYC were observed particularly at the PM using the PLA technique with vehicle (Veh) treatment (top panel). Reduced binding was evident after PP1 treatment (2 μM) for 24 hours in the bottom representative panel. D, Iodide uptake in TPC1 cells transfected with VO or NIS and treated with either vehicle or PP1 (2 μM) for 24 hours. NIS-mediated iodide uptake was demonstrated through the use of controls treated with 100 μM sodium perchlorate (NaClO_4_) for 1 hour before addition of ^125^I. E, Western blot analysis of endogenous NIS expression in human primary thyroid cultures treated for 24 hours with either vehicle or PP1 (2 μM). F, Human primary thyroid cells were transfected with VO or PBF-HA, and 48 hours later iodide uptake assays were performed. A significant reduction in iodide uptake was observed with PBF overexpression. Cultures treated with PP1 (2 μM) for 24 hours before the addition of ^125^I demonstrated uptake comparable with that of VO-transfected cells. n = 6 cultures. ns, not significant. ***, *P* < .001; **, *P* < .01; *, *P* < .05.

Because PBF binds NIS and alters its subcellular localization (15), we next examined the relevance of the Y174 site to NIS interaction. Coimmunoprecipitation studies in COS-7 cells revealed that alanine substitution at the Y174 residue almost completely abrogated PBF binding to NIS (Figure 5A). The interaction between NIS and PBF-HA was also observed using the PLA. Once again, the Y174A mutant demonstrated a very significantly reduced ability to bind NIS. Thus, the Y174 residue is critical for the interaction between PBF and NIS. Given the observed colocalization between PBF-pY174 and NIS, we then assessed whether the phosphorylated form of PBF could bind NIS. An interaction was detected between pY174 and NIS by coimmunoprecipitation after the transfection of PBF-HA and NIS-MYC in HeLa cells (Figure 5B). Binding between pY174 and NIS was also demonstrated in COS-7 cells using the PLA (Figure 5B). We then used the PLA technique within this context to assess the effect of Src inhibition on the interaction between PBF-pY174 and NIS. Sites of interaction between PBF-pY174 and NIS were once again observed, with the most intense areas of staining apparent at the PM (Figure 5C). After PP1 treatment, there appeared to be a reduction in both the overall amount of binding and in the level of interaction at the PM (Figure 5C). These observations suggest that the phosphorylated form of PBF can bind NIS, at least partly at the PM, and that inhibition of Src with PP1 can diminish this interaction.

### Treatment with PP1 overcomes PBF repression of iodide uptake

PBF has been shown to potently repress iodide uptake in vitro and in vivo (15, 16). Oncogenic expression would therefore be hypothesized to result in inappropriate retention of NIS away from the PM and hence reduced efficacy of radioiodine ablation therapy. Given the importance of Y174 to PBF:NIS binding and colocalization, we finally determined the influence of modulating PBF phosphorylation status on iodide uptake. TPC1 cells demonstrated no NIS-specific endogenous iodide uptake compared with that for sodium perchlorate-treated controls (Figure 5D). Transfection with NIS resulted in a significant increase in NIS-mediated uptake (1.16 ± 0.04 vs 0.17 ± 0.01 pmol of I^−^ per μg of protein compared with values for VO treatment, *P* < .001, n = 8) (Figure 5D). In cells containing exogenous NIS, uptake could be significantly enhanced through PP1 treatment (1.93 ± 0.19 vs 1.16 ± 0.04 pmol of I^−^ per μg of protein compared with values for vehicle treatment, *P* = .007, n = 8) (Figure 5D). Treatment with PP1 did not elicit an increase in endogenous iodide uptake, which may suggest that Src inhibition cannot stimulate NIS expression but instead induces NIS activity in a posttranslational manner.

Treatment of human primary thyroid cultures with PP1 (2 μM) for 24 hours also confirmed no effect on endogenous NIS protein expression levels (0.96 ± 0.07-fold change compared with that for vehicle treatment, *P* = .656, n = 3) (Figure 5E). As shown previously in human primary thyroid cells (14), PBF-HA overexpression was associated with a significant decrease in ^125^I uptake (47.2 ± 9.2% reduction, *P* = .004, n = 6) (Figure 5F). However, treatment of PBF-HA–transfected cultures for 24 hours with 2 μM PP1 entirely rescued iodide uptake (1.82 ± 0.42-fold increase compared with PBF-HA and vehicle treatment, *P* = .048, n = 6) (Figure 5F). This resulting uptake in PBF-HA-transfected cells treated with PP1 was not significantly different from that for untransfected, vehicle-treated cells (0.96 ± 0.2-fold, *P* = .848, n = 6) (Figure 5F). Hence, PP1 treatment completely abrogated the repression of iodide uptake by PBF. These data suggest, therefore, that overcoming PBF repression of NIS may be possible by targeting its phosphorylation by Src.

## Discussion

For the first time, these data identify the proto-oncogene PBF as a phosphoprotein and highlight the importance of the tyrosine residue Y174 in both the endocytosis of PBF and its interaction with NIS. Critically, we have associated the Src tyrosine kinase with phosphorylation of PBF Y174 and established that, through reduction of pY174 levels, PBF repression of iodide uptake can be ameliorated. Furthermore, the effect of PBF overexpression on NIS localization appears to be ubiquitous, suggesting that therapeutic targeting of PBF will be required for efficacious radioiodine uptake in tumors with high PBF expression. This would apply equally to tumors with endogenous NIS expression such as thyroid or breast cancer, to tumors in which NIS expression may be induced, such as poorly differentiated or anaplastic thyroid cancer, and to tumors in which NIS gene therapy may be administered, such as prostate or ovarian cancer.

Although radioiodine is generally considered to be a safe treatment modality, risks of a second malignancy and other adverse effects indicate that administered radiation doses should be minimized, the gold standard being successful remnant ablation or treatment of metastatic disease with minimal doses. We propose that targeting PBF phosphorylation may in the future satisfy the dual goals of driving ^131^I efficacy with minimized radiation exposure.

The interaction of PBF with Src was initially identified by mass spectrometry and subsequently confirmed through GST pulldown assays and both forward and reverse coimmunoprecipitation assays. Although Src binds PBF and overexpression of Src is associated with increased Y174 phosphorylation, it is possible that the influence of Src on PBF is indirect and that Src interaction fulfills some other as yet unknown function. It is also conceivable that kinases that lie downstream of Src may be more specific targets to ameliorate PBF phosphorylation. Future studies will thus seek to identify the regions of PBF and Src necessary for interaction and will elucidate whether Src binding results directly in PBF phosphorylation.

Although there was broad colocalization of PBF with NIS in a panel of 10 distinct cell lines, clearly only a proportion of cytoplasmic and membranous protein binds and colocalizes at any particular moment. Notably, a direct interaction between NIS and the phosphorylated form of PBF was detected by 2 different methodologies and in both HeLa and COS-7 cells. However, we are unable at present to answer the question of precisely how phosphorylation of Y174 affects PBF localization, which will form the basis of future studies. PP1 inhibition of pY174 was potent and reproducible in untransformed human primary thyroid cells. This physiologically relevant model also revealed that PP1 was able to rescue PBF inhibition of radioiodine uptake. It is possible that these data underestimate the potency of PBF repression of NIS function, given that transfection efficiency for these primary cells, as we have previously reported (14), is in the order of 45% to 95%.

Because our recent data show that PBF also modulates the function of the monocarboxylate transporter 8 (17), which regulates thyroid hormone efflux from the thyroid gland (24, 25), our collective findings suggest a fundamental role for PBF in normal thyroid physiology, albeit one that appears to be dysregulated in neoplasia. The precise contribution of Y174 phosphorylation to the regulation of iodide uptake and thyroid hormone efflux in untransformed thyroid cells, however, remains to be discerned.

Overall, we propose tyrosine phosphorylation of PBF as a novel therapeutic target to overcome inhibition of radioiodine uptake. Further studies will now be conducted to seek to elucidate the precise mechanisms that regulate the interaction between PBF and NIS and to identify the most effective and specific inhibitor of PBF Y174 phosphorylation.

## References

[1] Dai G Levy O Carrasco N. Cloning and characterization of the thyroid iodide transporter. Nature. 1996;379:458–460 8559252 10.1038/379458a0

[2] Smanik PA Liu Q Furminger TL. Cloning of the human sodium iodide symporter. Biochem Biophys Res Commun. 1996;226:339–345 8806637 10.1006/bbrc.1996.1358

[3] Kogai T Taki K Brent GA. Enhancement of sodium/iodide symporter expression in thyroid and breast cancer. Endocr Relat Cancer. 2006;13:797–826 16954431 10.1677/erc.1.01143

[4] Castro MR Bergert ER Beito TG. Monoclonal antibodies against the human sodium iodide symporter: utility for immunocytochemistry of thyroid cancer. J Endocrinol. 1999;163:495–504 10588823 10.1677/joe.0.1630495

[5] Dohán O Baloch Z Bánrévi Z Livolsi V Carrasco N. Rapid communication: predominant intracellular overexpression of the Na^+^/I^−^ symporter (NIS) in a large sampling of thyroid cancer cases. J Clin Endocrinol Metab. 2001;86:2697–2700 11397873 10.1210/jcem.86.6.7746

[6] Wapnir IL van de Rijn M Nowels K. Immunohistochemical profile of the sodium/iodide symporter in thyroid, breast, and other carcinomas using high density tissue microarrays and conventional sections. J Clin Endocrinol Metab. 2003;88:1880–1888 12679487 10.1210/jc.2002-021544

[7] Tazebay UH Wapnir IL Levy O. The mammary gland iodide transporter is expressed during lactation and in breast cancer. Nat Med. 2000;6:871–878 10932223 10.1038/78630

[8] Riesco-Eizaguirre G Santisteban P. A perspective view of sodium iodide symporter research and its clinical implications. Eur J Endocrinol. 2006;155:495–512 16990649 10.1530/eje.1.02257

[9] Yaspo ML Aaltonen J Horelli-Kuitunen N Peltonen L Lehrach H. Cloning of a novel human putative type Ia integral membrane protein mapping to 21q22.3. Genomics. 1998;49:133–136 9570958 10.1006/geno.1998.5217

[10] Stratford AL Boelaert K Tannahill LA. Pituitary tumor transforming gene binding factor: a novel transforming gene in thyroid tumorigenesis. J Clin Endocrinol Metab. 2005;90:4341–4349 15886233 10.1210/jc.2005-0523

[11] Watkins RJ Read ML Smith VE. Pituitary tumor transforming gene binding factor: a new gene in breast cancer. Cancer Res. 2010;70:3739–3749 20406982 10.1158/0008-5472.CAN-09-3531 PMC2875163

[12] Boelaert K McCabe CJ Tannahill LA. Pituitary tumor transforming gene and fibroblast growth factor-2 expression: potential prognostic indicators in differentiated thyroid cancer. J Clin Endocrinol Metab. 2003;88:2341–2347 12727994 10.1210/jc.2002-021113

[13] Hsueh C Lin JD Chang YS. Prognostic significance of pituitary tumour-transforming gene-binding factor (PBF) expression in papillary thyroid carcinoma. Clin Endocrinol (Oxf). 2013;78:303–309 22888961 10.1111/cen.12007

[14] Boelaert K Smith VE Stratford AL. PTTG and PBF repress the human sodium iodide symporter. Oncogene. 2007;26:4344–4356 17297475 10.1038/sj.onc.1210221

[15] Smith VE Read ML Turnell AS. A novel mechanism of sodium iodide symporter repression in differentiated thyroid cancer. J Cell Sci. 2009;122:3393–3402 19706688 10.1242/jcs.045427 PMC2736868

[16] Read ML Lewy GD Fong JC. Proto-oncogene PBF/PTTG1IP regulates thyroid cell growth and represses radioiodide treatment. Cancer Res. 2011;71:6153–6164 21844185 10.1158/0008-5472.CAN-11-0720 PMC3184940

[17] Smith VE Read ML Turnell AS. PTTG-binding factor (PBF) is a novel regulator of the thyroid hormone transporter MCT8. Endocrinology. 2012;153:3526–3536 22535767 10.1210/en.2011-2030

[18] Kogai T Brent GA. The sodium iodide symporter (NIS): regulation and approaches to targeting for cancer therapeutics. Pharmacol Ther. 2012;135:355–370 22750642 10.1016/j.pharmthera.2012.06.007 PMC3408573

[19] Eggo MC King WJ Black EG Sheppard MC. Functional human thyroid cells and their insulin-like growth factor-binding proteins: regulation by thyrotropin, cyclic 3′,5′ adenosine monophosphate, and growth factors. J Clin Endocrinol Metab. 1996;81:3056–3062 8768874 10.1210/jcem.81.8.8768874

[20] Ambesi-Impiombato FS Parks LA Coon HG. Culture of hormone-dependent functional epithelial cells from rat thyroids. Proc Natl Acad Sci USA. 1980;77:3455–3459 6106191 10.1073/pnas.77.6.3455 PMC349635

[21] Hornbeck PV Kornhauser JM Tkachev S. PhosphoSitePlus: a comprehensive resource for investigating the structure and function of experimentally determined post-translational modifications in man and mouse. Nucleic Acids Res. 2012;40:D261–D270 22135298 10.1093/nar/gkr1122 PMC3245126

[22] Ohno H Fournier MC Poy G Bonifacino JS. Structural determinants of interaction of tyrosine-based sorting signals with the adaptor medium chains. J Biol Chem. 1996;271:29009–29015 8910552 10.1074/jbc.271.46.29009

[23] Schweppe RE Kerege AA French JD Sharma V Grzywa RL Haugen BR. Inhibition of Src with AZD0530 reveals the Src-Focal Adhesion kinase complex as a novel therapeutic target in papillary and anaplastic thyroid cancer. J Clin Endocrinol Metab. 2009;94:2199–2203 19293266 10.1210/jc.2008-2511 PMC2690419

[24] Di Cosmo C Liao XH Dumitrescu AM Philp NJ Weiss RE Refetoff S. Mice deficient in MCT8 reveal a mechanism regulating thyroid hormone secretion. J Clin Invest. 2010;120:3377–3388 20679730 10.1172/JCI42113 PMC2929715

[25] Trajkovic-Arsic M Müller J Darras VM. Impact of monocarboxylate transporter-8 deficiency on the hypothalamus-pituitary-thyroid axis in mice. Endocrinology. 2010;151:5053–5062 20702572 10.1210/en.2010-0593

